# Screening, Identification, and Whole-Genome Sequencing of Ferulic Acid Esterase-Producing Lactic Acid Bacteria from Sheep Rumen

**DOI:** 10.3390/microorganisms13061295

**Published:** 2025-05-31

**Authors:** Mingxin Qiu, Yong Chen, Lei Wang, Luyu Li, Xiao Zhang, Zhuang Ma, Jiancheng Liu

**Affiliations:** Research Center for Biofeed and Animal Gut Health, College of Animal Science, Xinjiang Agricultural University, Urumqi 830052, China; xjauqmx@163.com (M.Q.); xjauwl@163.com (L.W.); xjlly1@163.com (L.L.); 18139230168@163.com (X.Z.); 18242644528@163.com (Z.M.)

**Keywords:** lactic acid bacteria, rumen, feruloyl esterase, whole-genome sequencing, *Lactobacillus mucosae*, *Streptococcus equinus*, probiotic properties, safety

## Abstract

Ferulic acid esterase (FAE) plays an important role in plant fiber degradation by catalyzing the hydrolysis of lignocellulosic structures. FAE-producing lactic acid bacteria (LAB), as potential probiotics, can improve ruminant digestion and gut health. In this study, two LAB strains (Q2 and Q6) with FAE activity were isolated from sheep rumen. Based on 16S rDNA sequencing, they were identified as *Lactobacillus mucosae* and *Streptococcus equinus*, respectively. Compared to Q2, Q6 demonstrated higher enzyme production, lactic acid yield, broader carbohydrate utilization, and stronger antimicrobial activity. The whole genome sequencing revealed Q2 and Q6 possess genomes of 2.14 Mbp and 1.95 Mbp, with GC contents of 46.81% and 37.30%, respectively. Q2 and Q6 exhibited the highest average nucleotide identity (ANI) with *L. mucosae* DSM 13345 (97.30%) and *S. equinus* ATCC 33317 (97.92%), respectively. The strains harbored 2101 and 1928 predicted genes, including 1984 and 1837 coding sequences (CDSs), respectively. GO enrichment analysis showed the CDSs predominantly associated with membranes (or cells), catalytic activity, and metabolic processes. KEGG analysis revealed both strains enriched in metabolic pathways, with Q6 showing a notably higher number of proteins in the ABC transporters and quorum sensing than Q2. Carbohydrate-active enzymes database (CAZy) profiling identified 75 CAZymes in Q2 and 93 CAZymes in Q6, with each strain containing one novel *fae* gene. Safety assessment identified 1 and 33 pathogenic genes, along with 2 and 4 putative antimicrobial peptide genes, in Q2 and Q6, respectively. Notably, Q6 carried 12 virulence factor genes. These findings suggest Q2 exhibits a superior safety profile compared to Q6, indicating a higher probability of Q2 being an effective probiotic strain. In conclusion, both LAB strains produce FAE. *L. mucosae* Q2 demonstrates suitability as a direct-fed probiotic for livestock, while Q6 exhibits potential as a silage inoculant, though comprehensive safety evaluations are required prior to its application.

## 1. Introduction

Lignocellulose is primarily composed of cellulose, lignin, pectin, proteins, and aromatic compounds [1]. Lignin binds tightly with cellulose and hemicellulose, strengthening plant cell walls but also creating barriers to microbial breakdown [2]. Ferulic acid (FA), a key phenolic compound [3], reduces digestibility by forming crosslinks between arabinoxylan and lignin via ester/ether bonds [4,5].

Ferulic acid esterase (FAE, EC 3.1.1.73), a subclass of carbohydrate esterases (CE), cleaves ester linkages between FA and polysaccharides in cell walls and releases FA or its dimers. It has been used in feed industry, paper manufacturing, bioenergy, and biorefining [6,7,8]. The rumen of ruminants typically contains sufficient glucanase and xylanase; however, a deficiency in FAE represents a critical bottleneck for complete biodegradation of cellulose and hemicellulose, thereby making FAE potentially essential for roughage digestion in ruminants [9]. Previous studies demonstrate that treating roughage or diets with FAE alone or in combination with non-starch polysaccharide enzymes and phytase enhances plant fiber degradation, promotes FA liberation, and increases volatile fatty acids production [10,11,12]. Furthermore, FAE supplementation improved diet palatability and quality, consequently enhancing the growth performance of animals [13,14]. These findings underscore the significant positive impacts of FAE on feed and animal nutrition.

FAE-producing microorganisms predominantly include bacteria (*Clostridium*, *Streptomyces*, *Pseudomonas*, and *Lactobacillus*) and filamentous fungi (*Aspergillus*, *Penicillium*, *Neurospora*, and *Talaromyces*), though most characterized FAEs are fungal-derived [15]. Beneficial FAE-producing microbes serve dual purposes as feed additives by enhancing the degradation of agricultural by-products and supporting gut health. Lactic acid bacteria (LAB), key probiotic organisms that ferment carbohydrates to lactate, have recently attracted research attention for FAE production. Based on published literatures, 17 FAE-producing *Lactobacillus* strains (including *L. plantarum*, *L. johnsonii*, *L. farciminis*, *L. fermentum*, *L. brevis*, *L. buchneri*, *L. reuteri*, and *L. crispatus*) have been evaluated in silage trials. These strains demonstrate the ability to reduce contents of neutral detergent fiber and acid detergent fiber, enhance fiber degradation efficiency, and increase free FA concentration in silage, consequently improving animal production performance and health status [16]. These findings highlight the potential of FAE-producing LAB for improving lignocellulose degradation and feed quality.

Current research on FAE-producing bacteria primarily focuses on utilizing *Lactobacillus* strains to improve silage fermentation quality. Ruminants can efficiently use the cellulose in roughage, making it possible to isolate FAE-producing LAB from their rumen microbiota. The rumen of ruminants harbors diverse FAE-producing microorganisms. This study aims to isolate and characterize novel FAE-producing microbial strains from ovine rumen. Through comprehensive whole-genome sequencing, we obtain genomic insights to facilitate the exploration and utilization of FAE-producing microbial resources, thereby providing valuable references for future research in this field.

## 2. Materials and Methods

### 2.1. Reagents, Media, and Strains

Methyl ferulate, ethyl ferulate, and 3-(N-morpholino) propanesulfonic acid (MOPS) were purchased from Solarbio Science & Technology Co., Ltd. (Beijing, China). The de Man, Rogosa, and Sharpe (MRS) medium was obtained from Haibo Biotechnology Co., Ltd. (Qingdao, China). Luria-Bertani (LB) medium, the Ezup Column Bacterial Genomic DNA Isolation Kit, and Taq PCR Master Mix were acquired from Sangon Biotech Co., Ltd. (Shanghai, China). The QIAamp DNA Mini Kit was procured from QIAGEN (Hilden, Germany). The Lactate II reagent kit was purchased from Analox (Hammersmith, London, UK). Carbohydrate fermentation test tubes were purchased from Hangzhou Microbial Reagent Co., Ltd. (Hangzhou, China). *Escherichia coli* CVCC 1382, *Staphylococcus aureus* CVCC 2257, and *Salmonella pullorum* CVCC 525 were obtained from the National Center for Veterinary Culture Collection (Beijing, China). The FAE-producing screening agar medium and FAE induction medium were prepared according to the method reported by Donaghy et al. [17].

### 2.2. Screening of Ferulic Acid Esterase-Producing Lactic Acid Bacteria

Fresh rumen fluid was collected from healthy adult Small-Tailed Han sheep fitted with permanent rumen cannulas. A 100 μL aliquot of rumen fluid was inoculated into 10.0 mL of MRS broth, sealed, and statically incubated at 37 °C for 12 h in an electric heating incubator (DHP-9162, Yiheng Scientific Instruments, Shanghai, China). The enriched culture was streaked onto FAE-producing LAB screening agar medium and incubated anaerobically (93% N_2_, 7% CO_2_) at 37 °C for 72 h in an anaerobic workstation (Defendor AMW 1000, Hariolab, Guangzhou, China). FAE-producing colonies were identified by the formation of distinct hydrolysis zones resulting from ethyl ferulate degradation. Isolates exhibiting clear zones were selected as potential FAE-producing LAB candidates.

For secondary screening, candidate strains were streaked onto fresh screening agar medium and incubated anaerobically at 37 °C for 72 h. Single colonies with clear zones were inoculated into MRS broth and incubated statically at 37 °C under anaerobic conditions for 12 h. Bacterial cells were harvested by centrifugation at 8000× *g* for 5 min (Minispin plus, Eppendorf AG, Hamburg, Germany), washed three times with sterile saline, and resuspended in deionized water to achieve OD_600_ of 0.8. Sterile Oxford cups (Φ 8 × 6 × 10 mm) were placed on FAE-screening agar plates, and 200 μL of bacterial suspension was added to each cup. After 72 h of dark incubation at 37 °C, diameters of clear halos were measured using an automated colony counter (ProtoCOL3 P3 HD/1129, Synbiosis, Cambridge, UK). Triplicates were used in three independent assays (*n* = 3).

### 2.3. Determination of FAE Activity

The candidate single colonies cultured overnight were harvested, washed, and resuspended in deionized water. The cell suspension (OD_600_ 0.8) was inoculated (2% *v*/*v*) into FAE induction medium and incubated anaerobically at 37 °C for 48 h. After incubation, the culture was centrifuged at 8000× *g* for 5 min at 4 °C, and the supernatant was collected as crude enzyme.

FAE activity was determined according to a modified method of Yue et al. [18]. In a 96-well UV-transparent microplate (Corning, NY, USA), 100 μL of 6.25-fold diluted crude enzyme was pre-incubated at 39 °C for 15 min. For the reaction tube, 200 μL of pre-warmed substrate solution (methyl ferulate in 100 mmol/L MOPS buffer, pH 6.5, at a final concentration of 100 μmol/L) was added. The blank tube received 200 μL of 100 mmol/L MOPS buffer. After 30 min incubation at 39 °C, absorbance at 340 nm (OD_340_) was measured before and after reaction using a microplate reader (Infinite M200, Tecan, Männedorf, Switzerland). Three independent enzyme activity assays were performed in duplicate reactions (n = 3). Enzyme activity (U/mL) is calculated as follows:

(1) $$\mathrm{FAE}\;\mathrm{activity}\;(U/\mathrm{mL})=\frac{\left(\Delta OD_{340,sample}-\Delta OD_{340,blank}\right)\times V_{total}\times DF}{t\times \left(l\times \varepsilon_{methyl\;ferulate}-l\times \varepsilon_{FA}\right)\times v_{sample}}$$

where ΔOD_340, sample_ = OD_340, initial_ − OD_340, final_ (reaction tube); ΔOD_340, blank_ = OD_340, initial_ − OD_340, final_ (blank tube); V_total_ is total reaction volume (0.3 mL); DF is dilution factor (6.25); t is reaction time (30 min); l is the pathlength (cm); ε_methyl ferulate_ is molar extinction coefficient of MF; ε_FA_ is molar extinction coefficient of FA; and v_sample_ is sample volume (0.1 mL).

One unit of enzyme activity was defined as the amount of enzyme releasing 1.0 μmol of FA/(min·mL) under the conditions described above.

### 2.4. 16S rDNA Identification

Purified candidate strains were subcultured three times before genomic DNA extraction using the Ezup Column Bacterial Genomic DNA Isolation Kit [19]. The full-length 16S rDNA was amplified by PCR with universal primers 27F (5′-AGAGTTTGATCMTGGCTCAG-3′) and 1492R (5′-CGGTTACCTTGTTACGACTT-3′) [20] using the following reaction system: 2.5 μL 10×Taq PCR Master Mix, 1.0 μL each of 10 μmol/L primers, 1.0 μL DNA template, and ddH_2_O to a final volume of 25.0 μL on a thermal cycler (PCR-96, BBI Lifesciences, Shanghai, China). Thermal cycling conditions comprised 95 °C for 5 min; 30 cycles of 94 °C for 30 s, 57 °C for 30 s, and 72 °C for 30 s; followed by 72 °C for 10 min. Amplicons were sequenced by Sangon Biotech using a 3730XL DNA Analyzer (Applied Biosystems, CA, USA). Sequences were aligned against the GenBank database via NCBI BLAST 2.14.0+ (https://blast.ncbi.nlm.nih.gov/Blast.cgi (accessed on 7 November 2023)) for taxonomic identification. A neighbor-joining phylogenetic tree was constructed using MEGA 11 [21].

### 2.5. Carbohydrate Fermentation Test

One loopful of bacterial suspension was transferred into carbohydrate fermentation test tubes containing esculin, cellobiose, maltose, mannitol, salicin, sorbitol, sucrose, raffinose, inulin, or lactose. The mixture was then thoroughly mixed and incubated anaerobically at 37 °C for 72 h. The results (positive or negative) were assessed according to the manufacturer’s guidelines based on observed color changes.

### 2.6. Growth Kinetics and Lactate Production

Growth curves were generated using a transparent flat-bottom 96-well microplate (Xinyou Biotechnology, Hangzhou, China). Each well contained 200 μL MRS broth inoculated with 2.5 μL bacterial suspension (OD_600_ 0.6–0.8). Following sealing, the plates were incubated anaerobically at 37 °C for 24 h. Bacterial growth was monitored by measuring OD_600_ every 2 h using a microbial growth analyzer (MGC-200; Scientz, Ningbo, China). Parallel sampling was performed at 2 h intervals, with 2 mL aliquots collected for pH measurement using a pH meter (FE20-Five Easy Plus, Mettler Toledo, Zurich, Switzerland) and lactate concentration determination with an LM5 analyzer (Analox, Hammersmith, London, UK).

### 2.7. Antimicrobial Activity

Overnight cultures of *E. coli*, *S. aureus*, and *S. pullorum* were inoculated into fresh LB broth and incubated until the OD_600_ reached 0.8. A volume of 0.1 mL of the pathogen suspension was thoroughly mixed with 15 mL of sterile LB agar (∼60 °C), and the mixture was immediately poured into sterilized Petri dishes. After solidification, Oxford cups were placed equidistantly on the agar surface, and 0.2 mL bacteria suspension of candidate strains (OD_600_ 0.8) was gently added to each cup. The bacteria suspension was allowed to diffuse on the medium for 12 h at 4 °C, after which the plates were incubated at 37 °C for 24 h, and the diameters of inhibition zone were measured using ProtoCOL3.

### 2.8. Whole Genome Sequencing and Bioinformatics Analysis

#### 2.8.1. Genomic DNA Extraction, Sequencing, Quality Control, and Assembly

The bacterial genomic DNA of candidate strains was extracted from 1.0 mL overnight cultures using the QIAamp DNA Mini Kit following the manufacturer’s protocol. DNA integrity was verified by 0.5% agarose gel electrophoresis, and concentration was quantified using a spectrophotometer (Nanodrop 2000, Thermo Fisher Scientific, Waltham, MA, USA) and a fluorometer (Qubit™ 3.0, Thermo Fisher Scientific, Waltham, MA, USA). SMRT Bell libraries were constructed with the SMRTbell Template Prep Kit (version 2.0) (Pacific Biosciences, CA, USA), quantified using Qubit™ 3.0, and assessed for insert fragment size using an Agilent 2100 Bioanalyzer System (Agilent Technologies, Santa Clara, CA, USA). Sequencing was performed by Biomarker Technologies (Wuhan, China) on the PacBio HiFi 30X platform (Pacific Biosciences, Menlo Park, CA, USA).

The sequencing raw data was processed with SMRT Link v11.0 to generate circular consensus sequencing reads. Following removal of reads < 2000 bp, high-quality clean reads were subjected to de novo assembly using Hifiasm (v0.19.5), with subsequent error correction performed by Pilon (v1.22) incorporating Illumina short-read sequencing data. Contigs were aligned against the NCBI nucleotide sequence database to determine chromosome types. Sequencing depth was calculated, as described by Rabha et al. [22], and genomes were circularized using Circlator v1.5.5.

#### 2.8.2. Bioinformatics Analysis

Coding sequences (CDSs) were predicted with Prodigal v2.6.3. Potential pseudogenes were analyzed using GenBlastA v1.0.4 and GeneWise v2.2.0. Average nucleotide identity (ANI) was calculated via the Maximal Unique Matches algorithm on the JSpeciesWS platform (https://jspecies.ribohost.com/jspeciesws (accessed on 26 March 2025)). tRNA genes were predicted using tRNAscan-SE v2.0, while ribosomal RNA (rRNA) genes were identified with Infernal v1.1.3. Repeat sequences were predicted using RepeatMasker v4.1.8, and genomic islands were identified using IslandPath-DIMOB v0.2. The presence of prophage sequences within the genome was assessed with PhiSpy v2.3, and CRISPR (clustered regularly interspaced palindromic repeats) elements were predicted using CRT v1.2. Secondary metabolite gene clusters were predicted using antiSMASH v7.0, and promoter regions were identified using PromPredict v1.0.

The functional annotation of the predicted proteins was conducted using Blast2GO v2.5, employing BLAST 2.14.0+ (E-value ≤ 1.00 × 10^−5^) against several databases, including the Non-Redundant Protein Sequence Database (Nr), Transporter Classification Database (TCDB), Gene Ontology (GO), Kyoto Encyclopedia of Genes and Genomes (KEGG), evolutionary genealogy of genes: Non-supervised Orthologous Groups (eggNOG), Pfam, SwissProt, and TrEMBL. The carbohydrate-active enzymes database (CAZy) was queried using HMMER v3.4 with hidden Markov models to identify carbohydrate hydrolases. SignalP v4.0 was utilized to predict the presence of signal peptides and their cleavage sites in proteins, while TMHMM v2.0 was employed to determine whether proteins are transmembrane proteins. Antibiotic resistance genes (ARGs) within the CDSs were searched using the Resistance Gene Identifier (RGI) from the Comprehensive Antibiotic Resistance Database (CARD 4.0) (https://card.mcmaster.ca (accessed on 28 March 2025)) [23], with predictive patterns selected under two modes, perfect and strict, identity ≥ 80%.

The virulence factor database (VFDB) (https://www.mgc.ac.cn/VFs/ (accessed on 28 March 2025)), Antimicrobial Peptide Database (dbAMP 3.0) (https://awi.cuhk.edu.cn/dbAMP/ (accessed on 28 March 2025)), and PHI-base 5.0 (http://www.phi-base.org/ (accessed on 27 March 2025)) were downloaded. Multi-sequence alignments were performed using TBTools-II software [24], with criteria set for screening virulence factor genes (VFGs) [25], potential antimicrobial peptides (AMPs) [26], and pathogenic genes (PGs) [27] based on an amino acid sequence identity ≥ 80%, coverage ≥ 90%, and E-value ≤ 1.00 × 10^−5^.

The probability of probiotics was predicted using iProbiotics (http://bioinfor.imu.edu.cn/iprobiotics/public/ (accessed on 20 March 2025)) [28]. The probiotic potential risk score (PPRS) for candidate strains was calculated through the ProbioMinServer platform (https://probiomindb.imst.nsysu.edu.tw/ (accessed on 28 March 2025)), with thresholds defined as low-risk (≤4), medium-risk (4–6), and high-risk (≥6) [29].

### 2.9. Data Analysis

Statistical analysis was performed using IBM SPSS Statistics 26.0 (IBM Corp., Armonk, NY, USA) and GraphPad Prism 9.0 (GraphPad Software, San Diego, CA, USA). One-way analysis of variance was conducted for group comparisons. Data are presented as mean and standard deviation. Statistical significance was set at *p* < 0.05.

## 3. Results

### 3.1. Screening of FAE-Producing Strains

**Figure 1:**
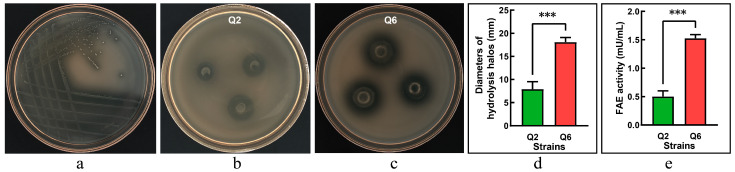
Screening of ferulic acid esterase (FAE)-producing lactic acid bacteria. (**a**): candidate strains forming hydrolysis zones on FAE-screening agar. (**b**): clear halos produced by the strain Q2 in Oxford cup assays. (**c**): clear halos produced by the strain Q6 in Oxford cup assays. (**d**): diameters of hydrolysis halos (excluding Oxford cup diameter: Φ8.0 mm). (**e**): FAE activity. Values are mean and standard deviation (*n* = 3). *** *p* < 0.001.

Following enrichment in MRS broth, rumen microbiota was streaked onto FAE-screening agar plates, resulting in distinct clear zones surrounding candidate colonies (Figure 1a). The purified isolates were further validated using the Oxford cup assay, where two strains, Q2 and Q6, produced measurable hydrolysis halos (Figure 1b,c). The strain Q6 exhibited a significantly larger halo diameter compared to the strain Q2 (*p* < 0.001, Figure 1d), indicating a superior capacity for ethyl ferulate degradation. FAE activity assays confirmed both strains produced functional enzymes; however, Q6 showed significantly higher activity than Q2 (*p* < 0.001, Figure 1e).

### 3.2. 16S rDNA Sequence Alignment

The strains most similar to Q2 and Q6 based on 16S rDNA sequence alignment are presented in Table 1. The sequence comparison reveals that Q2 shares 100% identity with *Lactobacillus mucosae* JCM 12515. It is noteworthy that *L. mucosae* has been referred to as *Limosilactobacillus mucosae* [30].

**Figure 2:**
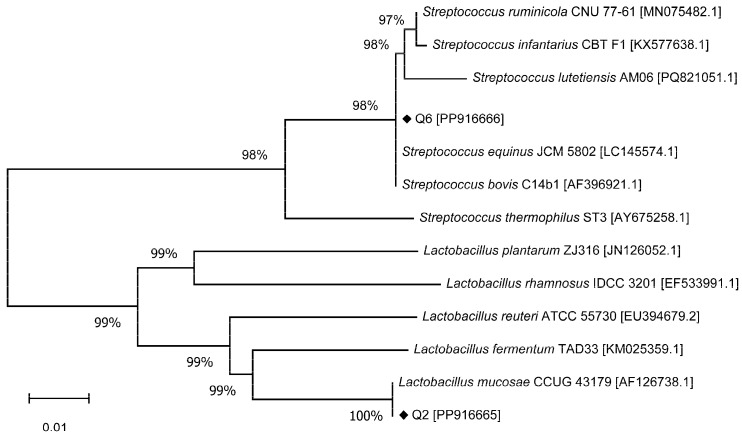
Phylogenetic tree of bacterial strains based on 16S rDNA sequences.

In the case of Q6, it exhibited 100% identity with *Streptococcus equinus* JCM 5802 and *Streptococcus bovis* C14b1. The phylogenetic tree depicting Q2 and Q6 in relation to several known bacterial species is illustrated in Figure 2. Aside from *L. mucosae*, Q2 also shared 94.72% and 94.98% identity to *L. reuteri* and *L. fermentum*, respectively. Q6 demonstrated 99.86% identity to both *S. ruminicola* and *S. infantarius* in addition to its matches with *S. equinus* and *S. bovis*. Based on these results, Q2 was identified as *L. mucosae*. Because *S. bovis* and *S. equinus* are considered synonymous, and since *S. equinus* was the first to be described, it is currently prioritized [31]. Consequently, Q6 was identified as *S. equinus*. The 16S rDNA sequences have been deposited in GenBank under accession numbers PP916665 (Q2) and PP916666 (Q6).

### 3.3. Carbohydrate Utilization

Table 2 presents the results of acid production from carbohydrate fermentation by Q2 and Q6. As indicated in Table 2, Q2 effectively fermented maltose, sucrose, and raffinose and exhibited positive reactions. Conversely, it was unable to ferment sculin, cellobiose, mannitol, salicin, sorbitol, inulin, and lactose, resulting in negative reactions. In contrast, Q6 showed a negative reaction solely to mannitol among all tested carbohydrates, while demonstrating positive reactions to all other carbon sources.

### 3.4. Growth Curves, pH Dynamics and Lactate Production Profiles

**Figure 3:**
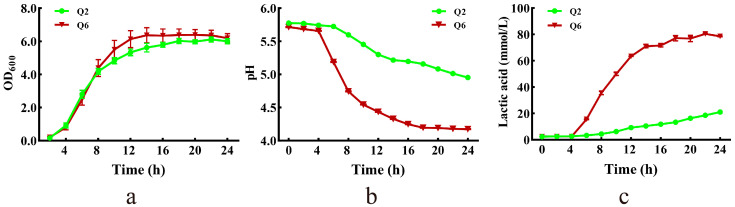
Growth curve, and lactic acid production of Q2 and Q6. (**a**): growth curves. (**b**): pH dynamics of culture. (**c**): lactate production profiles. Values are mean and standard deviation (*n* = 3).

The growth curves, pH dynamics, and lactate production profiles of Q2 and Q6 are presented in Figure 3. Both strains grew slowly during the lag phase (0–4 h). Q6 reached stationary phase earlier (14 h vs. 18 h for Q2) (Figure 3a). Concurrent pH decline correlated with lactate accumulation—a medium pH of Q6 dropped to 4.2 by 18 h, while Q2 reached only pH 5.0 by 24 h (Figure 3b). Q6 exhibited faster lactate production, stabilizing at 78 mmol/L by 18 h; whereas, Q2 produced merely 21 mmol/L by the end of incubation (Figure 3c).

### 3.5. Antimicrobial Activity Against Pathogenic Bacteria

**Figure 4:**
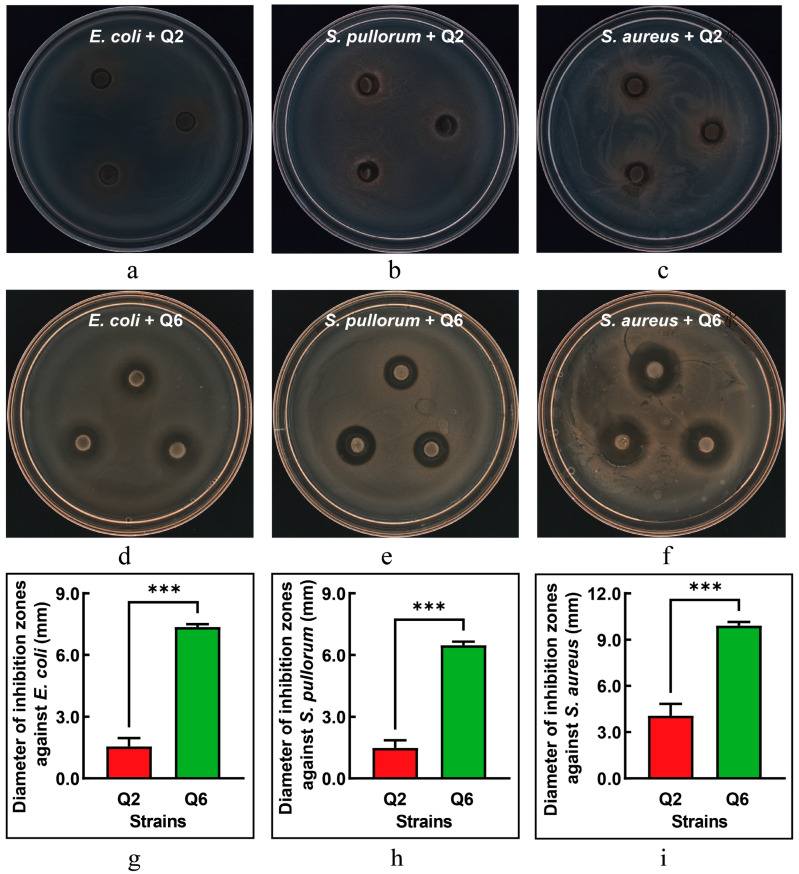
The inhibitory effect of Q2 and Q6 against pathogenic bacteria. (**a**–**c**): inhibition zones of Q2 against *E. coli*, *S. pullorum*, and *S. aureus*, respectively. (**d**–**f**): inhibition zones of Q6 against the same pathogens. (**g**–**i**): Q6 exhibited significantly stronger inhibitory effects against all three pathogens compared to Q2. Values are mean and standard deviation (*n* = 3). *** *p* < 0.001.

The antibacterial activities of Q2 and Q6 against *E. coli*, *S. pullorum*, and *S. aureus* are illustrated in Figure 4. Q2 exhibited only mild antibacterial effects against *E. coli* and *S. pullorum* (Figure 4a,b), while its antibacterial activity against *S. aureus* showed a slight increase (Figure 4c). In contrast, Q6 demonstrated significant antibacterial effects against all three pathogenic bacteria (Figure 4d–f), with *S. aureus* being the most sensitive to Q6. The inhibitory effects of Q6 on the three pathogenic bacteria were significantly higher than those of Q2 (Figure 4g–i).

### 3.6. Whole Genome Sequencing

#### 3.6.1. Genome Sequencing, Assembly, and Gene Prediction

**Figure 5:**
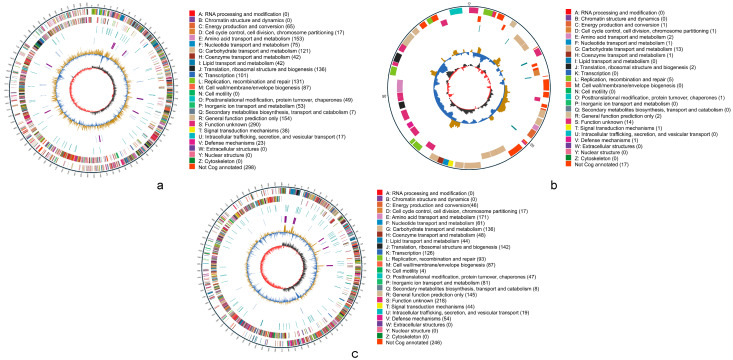
Circular genome maps of Q2 and Q6, generated using Circos v0.66 based on COGs (Clusters of Orthologous Groups) annotation results. (**a**): chromosome of Q2. (**b**): plasmid of Q2. (**c**): chromosome of Q6. The outermost circle represents the scale of the genome size, with each tick mark indicating 5 kb. The second and third circles represent genes on the forward and reverse strands of the genome, respectively. Different colors indicate different COGs’ (Clusters of Orthologous Groups) functional categories. The fourth circle represents repetitive sequences. The fifth circle shows tRNA and rRNA, where blue indicates tRNA and purple indicates rRNA. The sixth circle displays the GC content. The light-yellow regions indicate areas where the GC content is higher than the average GC content of the genome, while the blue regions indicate areas where the GC content is lower than the average GC content of the genome. The height of the peaks reflects the degree of deviation from the average GC content, with higher peaks indicating greater differences. The innermost circle represents GC-skew, where dark gray regions indicate areas with G content greater than C, and red regions indicate areas with C content greater than G.

The basic information of the sequencing data is presented in Table S1. After quality control, the clean reads for Q2 and Q6 were 61,469 and 35,503, respectively, with the distribution of read lengths shown in Figure S1. Following assembly, the key features of Q2 and Q6 genomes are summarized in Table 3, with the genome assembly results in Table S2 and the contig classification information in Table S3. The genome of Q2 consisted of a circular chromosome measuring 2,068,302 bp (Figure 5a) and a plasmid of 68,038 bp (Figure 5b), with a sequencing depth of 156× and a GC content of 46.81%. In contrast, Q6 comprised a single circular chromosome of 1,950,612 bp (Figure 5c), with a sequencing depth and GC content of 144× and 37.30%, respectively. The ANI of the Q2 strain with the reference genome *L. mucosae* DSM 13345 (Genome ID: GCA_001436025.1) was 97.30%. For Q6, the ANI with *S. equinus* ATCC 33317 (Genome ID: GCA_000747195.1) was 97.92%. The genome annotation revealed that Q2 harbors a total of 2101 genes, including 1984 CDSs, which accounted for 94.43% of all predicted genes and 117 RNA genes. For Q6, a total of 1928 genes were predicted, comprising 1837 CDS (95.28% of all genes) and 91 RNA genes. Genomic components are detailed in Table S4. The genome sequences have been deposited in GenBank with accession nos. GCA_049562395.1 (Q2) and GCA_049560825.1 (Q6).

#### 3.6.2. Gene Annotation in Universal Databases

The numbers of annotated genes in the universal databases for CDSs are shown in Table 4. The Q2 strain contains a total of 1967 annotated CDSs, representing 99.14% of the total CDSs, while the Q6 strain has 1831 annotated CDSs, accounting for 99.67% of the total CDSs.

**Figure 6:**
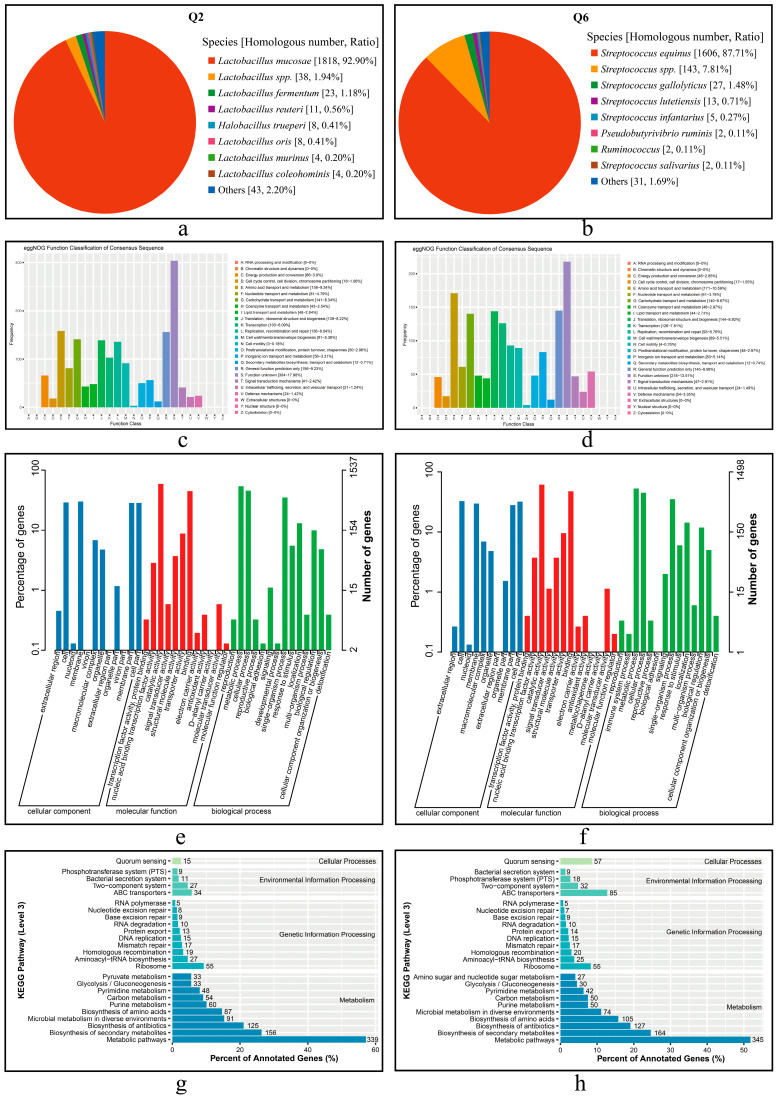
Functional annotation of predicted CDSs and the proteins in Q2 and Q6. (**a**,**b**): Nr annotation of Q2 and Q6, respectively. (**c**,**d**): eggNOG annotation of Q2 and Q6, respectively. (**e**,**f**): GO annotation of Q2 and Q6, respectively. (**g**,**h**): KEGG annotation of Q2 and Q6, respectively.

The annotation results of proteins from Q2 and Q6 in the Nr database are presented in Figure 6a,b. A total of 1957 proteins of Q2 were annotated to *L. mucosae*, accounting for 92.90% of the total putative proteins (Figure 6a). For Q6, 1831 proteins were annotated, of which 87.71% were assigned to *S. equinus*, and 7.81% were annotated to *Streptococcus* spp. (Figure 6b).

The annotation results of CDSs from Q2 and Q6 in the eggNOG database are shown in Figure 6c,d. Q2 matched a total of 1669 genes (1625 chromosomal and 45 plasmid- translated), while Q6 matched 1591 genes. Among these, functional unknowns accounted for 18.21% in Q2 and 13.70% in Q6. Dominant functional classes included amino acid transport and metabolism (Q2:158; Q6:171), carbohydrate transport and metabolism (141 vs. 140), and translation, ribosomal structure, and biogenesis (139 vs. 144).

The GO annotation results are presented in Figure 6e,f. The GO database comprises the following three categories: cellular component (CC), molecular function (MF), and biological process (BP). Comparative analysis revealed that in CC, membrane and cell -related terms were predominant (Q2: 461 [29.99%]; Q6 489 [32.64%]); both strains shared catalytic activity dominance in MF (Q2: 907 [59.01%]; Q6: 919 [61.35%]), and metabolic processes constituted >52% of BP annotations (Q2: 829 [53.94%]; Q6: 792 [52.87%]).

The KEGG pathway annotation results are shown in Figure 6g,h. The CDSs from both strains were primarily enriched in the following four KEGG pathway (level 1) classes: metabolic pathways, ribosome, ABC transporters, and quorum sensing, with the highest number of CDSs found in metabolic pathways. Notably, substantial inter-strain differences were observed. Q6 demonstrated significantly greater representation in both ABC transporters (85 vs. 34 in Q2) and quorum sensing pathways (57 vs. 15 in Q2), suggesting distinct functional specialization between these strains.

#### 3.6.3. Gene Annotation in Special Databases

**Figure 7:**
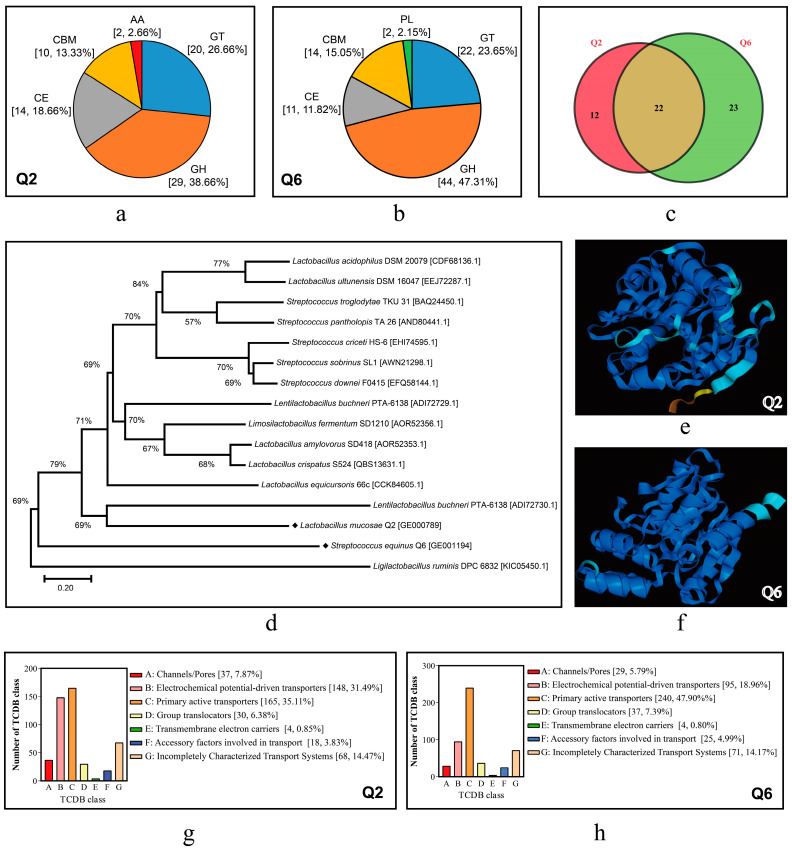
CDSs annotated in CAZy and TCDB databases and FAEs of Q2 and Q6. (**a**,**b**): distribution profiles of carbohydrate-active enzymes in Q2 and Q6. GH, glycoside hydrolases; GT, glycosyl transferases; CE, carbohydrate esterases; AA, auxiliary activities; CBM, carbohydrate-binding modules; PL, polysaccharide lyases. (**c**): Venn diagram illustrating shared and unique CAZyme family members between Q2 and Q6. (**d**): phylogenetic tree of FAEs. (**e**,**f**): predicted three-dimensional structures of FAEs derived from Q2 and Q6. (**g**,**h**): CDSs annotated to the TCDB in Q2 and Q6.

The genome annotation results of Q2 and Q6 in several special databases are summarized in Table 5. CAZy analysis identified 75 (3.78% of CDSs) and 93 (5.06%) carbohydrate-active enzymes (CAZymes) in Q2 and Q6 respectively, with glycoside hydrolases (GHs) predominant (38.66% Q2 vs. 47.31% Q6) followed by glycosyl transferases (GTs; 26.66% vs. 23.65%). Strain-specific patterns emerged, such as exclusive auxiliary activities (AAs) in Q2 (Figure 7a) and polysaccharide lyases (PLs) in Q6 (Figure 7b), while shared 22 CAZyme families contrasted with unique expansions (12 families/35.29% in Q2 vs. 23/48.89% in Q6; Figure 7c).

Genomic analysis of GHs revealed distinct profiles between the strains. Q2 harbored 5 α-amylase genes, while Q6 possessed six copies. Both strains encoded oligosaccharide-degrading enzymes (α/β-galactosidases, β-glucuronidase, β-fructosidase, β-glucosidase) and polysaccharide-active xylanase and cellulases. Q6 exhibited significantly higher gene copy numbers for both simple and complex CAZymes. This indicates that Q6 has superior starch, cellulose, and hemicellulose hydrolysis potential compared to Q2. A single FAE gene was identified in both Q2 and Q6 genomes (for the nucleotide and amino acid sequences, see Table S5). Phylogenetically, the FAE of Q2 (GE000789) was relatively close to that of *Lentilactobacillus buchneri*, while the FAE of Q6 (GE001194) was formed a distinct clade separate from characterized Streptococcus FAEs (Figure 7d). Additionally, there are significant differences in their 3D structures (Figure 7e,f).

Analysis of the key gene for lactic acid production, the lactate dehydrogenase gene (*ldh*), revealed that the genome of Q2 possessed 5 L-lactate dehydrogenase (*ldhL*) genes and 3 D-lactate dehydrogenase (*ldhD*) genes, while only 2 *ldhL* genes were found in the genome of Q6 (for details, see Table S6).

Using SignalP v4.0, a total of 81 and 106 proteins containing signal peptides were predicted in Q2 and Q6, respectively, along with 31 and 49 secretory proteins. Additionally, 470 and 501 transport-related proteins were identified in Q2 and Q6, respectively (Figure 7g,h). These proteins were predominantly enriched in subclasses such as primary active transporters and electrochemical potential-driven transporters.

The PHI-base annotation results are shown in Table S7. Comparative analysis revealed that only one PG, situated at the native chromosomal locus, was predicted in the Q2 genome, demonstrating high sequence identity (80.60%) with the *walR* gene of *Enterococcus faecalis*. In contrast, 33 PGs were identified in the Q6 genome, of which two were located within the prophage region, indicating that most PGs are intrinsic to the *S. equinus* species. Among these PGs, 20 were classified in the “reduced virulence” category, followed by “unaffected pathogenicity”. The “increased virulence” category represents critical pathogenic genes, with only one PG identified in Q2 compared to three PGs in Q6 (for details, see Table S7).

Employing RGI analysis, no ARGs were found in either Q2 or Q6. Based on VFDB, no potential VFG was identified in Q2, while 12 putative VFGs were annotated in Q6. Among these, 6 VFGs were associated with immune modulation, and 2 exhibited exotoxin-related functions (for details, see Table S8). According to dbAMP 3.0, two potential AMPs were predicted in the proteins encoded by Q2, while four AMPs were found in Q6 (for details, see Table S9). Additionally, an enterolysin A precursor gene was found in Q2. In Q6, there were two bovicin 255 precursor genes and five lantibiotic biosynthesis genes, involving in the synthesis and transport of Nisin. Using antiSMASH v7.0, three secondary metabolite gene clusters were predicted in Q6, of which two were related to the synthesis of surfactin and ubericin K. iProbiotics analysis classified Q2 as a high-probability probiotic candidate (85.82% general probiotic likelihood; 99.67% *Lactobacillus* genus specificity), while the probability for Q6 being a probiotic was only 1.44%. Strain Q2 exhibited a PPRS of 1.00 (low-risk), while Q6 showed a significantly higher PPRS of 30.41 (high-risk) (Table 5).

## 4. Discussion

*Lactobacillus*, *Streptococcus*, and *Bifidobacterium* constitute the primary LAB in the rumen of ruminants [32]. Their populations significantly increase when ruminants consume easily fermentable non-fiber carbohydrates (NFCs), such as grains [33]. In the rumen ecosystem, *Lactobacillus* comprises species such as *L. ruminis*, *L. acidophilus*, *L. fermentum*, *L. plantarum*, *L. buchneri*, *L. brevis*, *L. cellobiosus*, *L. salivarius*, etc. [34]. Although typically not the dominant microbial group, *Lactobacillus* becomes the predominant species in cattle experiencing acute ruminal acidosis. This ecological shift is closely related to lactic acid accumulation resulting from the rapid proliferation of *S. bovis*. This bacterium was first identified by Orla-Jensen [35] and later renamed *S. equinus* [36]. It is now recognized as part of the *Streptococcus bovis/Streptococcus equinus* complex (SBSEC) and commonly inhabits the digestive tracts of both humans and animals. Owing to its efficient starch utilization and rapid lactic acid production, *S. equinus* is considered a pathogen associated with severe acute ruminal acidosis and laminitis [37,38].

*Lactobacillus* is a widely used probiotic that can be employed as a direct-fed microbial to maintain intestinal health and improve animal production performance. *L. mucosae* was first identified in the pig small intestine and characterized by Roos et al. [39]. Subsequently, it was found in cattle [40], human [41], goat [42], sheep [43], and donkey [44].

In recent years, researchers have conducted genomic sequencing of *Lactobacillus mucosae* isolated from piglets [45], humans [46], and cattle [47], constructing both draft and complete genome maps. Current data reveal that the genome of *L. mucosae* shows a high identity with those of *L. reuteri* and *L. fermentum* [45]. According to the genomic data of all 15 *L. mucosae* strains available in the Bacterial and Viral Bioinformatics Resource Center (BV-BRC) (https://www.bv-brc.org/ (accessed on 30 March 2025)) [48], the genomes are approximately 2.10 Mbp in length (ranging from 1.88 Mbp to 2.37 Mbp), with an average GC content of 46.50% (ranging from 45.87% to 46.98%). The average number of CDSs is 2068 (ranging from 1883 to 2303), with an average of 75 tRNA genes (ranging from 41 to 103) and 13 rRNA genes (ranging from 2 to 37), along with varying numbers of plasmids. In the present study, the ANI between strain Q2 and *L. mucosae* DSM 13345 was 97.30%. Comparative genomic analysis using TBtools II reveals 135 proteins in Q2 that differ from those in DSM 13345. Of these, 63 are annotated as hypothetical proteins, while the remaining proteins with known functions exhibit functional roles in nucleic acid synthesis, carbohydrate hydrolysis, and phage-related processes. These findings suggest evolutionary differences among *L. mucosae* species in various habitats.

The genomic analysis of 13 *S. bovis* strains isolated from the digestive tracts and feces of sheep, goat, cattle, and horse, as recorded in the BV-BRC, reveals that the genome of *S. bovis* is approximately 1.86 Mbp in length (ranging from 1.62 Mbp to 2.00 Mbp), with a GC content of about 37.32% (ranging from 37.00% to 37.59%), and an average of 1813 CDSs (ranging from 1737 to 1987). The genome length of the Q6 strain isolated in this study is similar to that of *S. bovis* JB1 [49], with its GC content and CDS numbers falling within the aforementioned range. Although Q6 and *S. equinus* ATCC 33317 share a high ANI of 97.92%, Q6 encodes 165 proteins that show substantial differences from those in ATCC 33317. The comparison and analysis of the two genomes may help elucidate the metabolic differences between these two species.

The species epithet “*mucosae*” derives from its ability for intestinal mucosal adhesion, mediated by conserved mucus-binding proteins and adhesin-like proteins. The genome of *L. mucosae* DPC 6426 not only contains *mub* genes similar to other *L. mucosae* and *L. reuteri* strains but also harbors two collagen-binding proteins, one fibronectin-binding protein, and five LPXTG motif genes, all of which are associated with adhesion-related functions [47]. In the present study, a total of three *mub* genes, two adhesin-like protein genes, five LPXTG motif genes, and one collagen-binding protein gene were identified in the Q2 genome. These findings demonstrate that Q2 also possesses favorable mucosal adhesion capabilities and probiotic colonization potential.

Comparative genomic analyses of probiotic, pathogenic, and non-probiotic, non-pathogenic *E. faecium* strains reveal distinct genetic patterns; probiotic strains are enriched in carbohydrate metabolism-related genes; whereas, pathogenic strains predominantly carry replication/recombination genes—a divergence attributed to the probiotic requirement for utilizing diverse carbohydrates, while pathogens harbor more mobile element-associated replication/recombination genes [50]. In our study, both Q2 and Q6 encoded abundant carbohydrate hydrolases, with Q6 exhibiting a notably higher number of GH genes than Q2. GHs and GTs constituted the predominant CAZymes in both strains. These findings suggest that both Q2 and Q6 play active roles in carbohydrate digestion within the ruminant foregut, particularly in the breakdown of simple carbohydrates. Notably, the expanded GHs class in Q6 may reflect its adaptation to more complex carbohydrate substrates compared to Q2, potentially explaining their differential colonization capabilities within the rumen ecosystem.

Although both *L. mucosae* and *S. equinus* are LAB, their carbohydrate fermentation profiles differ significantly. Generally, *Lactobacillus* spp. typically ferments maltose, cellobiose, sucrose, lactose, and monosaccharides to produce lactic acid [34]. However, intestinal *L. mucosae* strains isolated from both domestic pigs and wild boars exhibited considerable strain-dependent variation in substrate utilization profiles [39,51]. In contrast, *S. equinus* ferments amygdalin, cellobiose, and arbutin but lacks the ability to metabolize maltose, ribose, or sorbitol [52]. In this study, compared to Q2, Q6 exhibited a notably broader substrate spectrum for acid production. This difference likely stems from genomic variations in CAZymes, highlighting the metabolic diversity of these strains in different ecological niches.

Although Q2 possesses more copies of *ldh* genes than Q6, our study found that Q6 exhibited higher acid production capacity than Q2. Several factors may contribute to this phenomenon. First, Q6 contains substantially more CAZymes, suggesting enhanced substrate supply for lactate biosynthesis. Second, while the *ldh* copy number is important, promoter architecture critically regulates expression. Comparative analysis of *Lachancea thermotolerans* strains revealed that single-nucleotide polymorphisms in promoter regions and distinct transcription factor binding sites correlate with high lactate production [53]. Notably, although Q2 possessed eight *ldh* gene copies, only four contain predicted promoters; whereas, both copies in Q6 were promoter-associated. Although this study did not compare the structures of these promoters, the findings indirectly suggest potential mechanisms underlying the differential acid production capacity. Third, the regulation of lactate production is related to various regulators. Yang et al. [54] identified glutamate racemase, methylated-DNA-protein-cysteine methyltransferase (MGMT), fumarate reductase flavoprotein subunit (frdA), and amidophosphoribosyltransferase as lactate production enhancers, while ribokinase, fructokinase, argininosuccinate lyase, and large subunit ribosomal protein L30 functioned as suppressors in *Lactobacillus panis* L7. Genomic analysis showed that Q2 contains all these genes (including two *frdA* copies but also four ribokinase and two fructokinase genes), while Q6 lacks both MGMT and ribokinase genes (see Table S10).

The rumen microbiome, particularly fungi and bacteria, serve as a reservoir for FAEs. Phylogenetic analysis of 15,293 FAE proteins showed that rumen FAEs are predominantly originated from anaerobic fungi, which display higher expression levels than bacterial FAEs [55]. Major rumen fungi involved in lignocellulose degradation include *Neocallimastix*, *Orpinomyces*, and *Piromyces*, while FAE-producing bacteria comprise *Butyrivibrio*, *Prevotella*, *Cellulosilyticum*, *Streptomyces*, and *Lactobacillus* [56]. Wong et al. [57] characterized seven FAE genes from bovine rumen metagenomes, with open reading frames ranging from 681 to 1359 bp, reflecting the considerable functional diversity of FAE in rumen. In this study, the two putative FAEs from the genomes of Q2 and Q6 shared low sequence identity with known FAEs, suggesting that they represent two novel FAEs.

*Lactobacillus*, *Enterococcus*, *Streptococcus*, and *Staphylococcus* represent the primary bacteriocin-producing microorganisms in animal intestines [58]. *Lactobacillus* predominantly synthesizes bacteriocins such as plantaricin, fermenticin, Nisin, gassericin, and pentocin, while *Streptococcus* produces Nisin H, thermophilin, and suicin [58], with *S. bovis* additionally generating bovicin [59]. To date, no bacteriocins have been isolated and characterized from *L. mucosae*. Research indicates that the mucus-binding proteins in *L. mucosae* LM1 exhibit antimicrobial activity through cell surface protection mechanisms [45]. Adhesin-like proteins may competitively bind to blood group antigen receptors in gastrointestinal mucosa, thereby preventing pathogen attachment [60]. The *L. mucosae* CRL573 genome harbors two potential enterolysin A genes [46], while the *L. mucosae* Marseille strain possesses 12 distinct bacteriocin genes coding 38 to 67 amino acids [61], demonstrating the abundance of bacteriocin-encoding genes in *L. mucosae*. In this study, we identified multiple genes’ coding potential for antimicrobial peptides or their precursors in the genomes of Q2 and Q6. Notably, Q6 contained not only the bovicin 255 gene but also gene clusters associated with surfactin and ubericin K biosynthesis. Surfactin represents a lipopeptide-class antimicrobial peptide [62], while ubericin K constitutes a novel pore-forming bacteriocin targeting the mannose phosphotransferase system [63]. These findings demonstrate that Q6 produces a more diverse array of antimicrobial peptides compared to Q2, explaining its superior antimicrobial activity. Importantly, the rich repertoire of bacteriocin genes in Q6 suggests its potential as a valuable resource for developing novel antimicrobial agents.

Safety evaluation is critical for the potential application of probiotic candidates. Bioinformatics safety assessments typically involve the prediction of ARGs, VFGs, PGs, as well as mobile genetic elements, like plasmid and phage-related genes [29]. VFGs directly mediate microbial pathogenicity. In the current study, comparative genome analysis identified one PG in Q2; whereas, Q6 harbored 12 VFGs and 33 PGs. These results suggest that Q6 may carry a much higher risk of causing infections and inflammatory responses, which may be the main reason for its extremely low probiotic probability and abnormally high PPRS score.

The SBSEC comprises seven (sub)species, which can be classified as human/animal commensals, emerging opportunistic pathogens, and food fermentation microorganisms. Some members are associated with various animal and human diseases, while others are considered beneficial for extending the shelf life and improving the quality of fermented foods [37]. Thus, SBSEC represents the only group that simultaneously includes both pathogenic strains and potential food-grade strains. Current research on SBSEC virulence factors primarily relies on homology-based identification with other *Streptococci* [37]. The safety and long-term effects of consuming large quantities of SBSEC members through food/feed remain unexplored.

Evidence indicates that *S. bovis* inoculation into silage feedstocks (e.g., Guinea grass, Tanzania grass, elephant grass, mulberry, alfalfa, and whole-plant triticale) significantly improves fermentation quality, enhances antioxidant capacity, increases the abundance of beneficial bacteria, and reduces nutrient loss in silage [64,65,66,67,68,69]. Some researchers suggest that *S. bovis/S. equinus* could potentially be used as silage inoculants [68,69,70]. However, it is important to note that, although *S. equinus* is a natural inhabitant of the rumen in ruminants, its safety must be rigorously and comprehensively evaluated before being approved as a silage inoculant.

## 5. Conclusions

This study successfully isolated two FAE-producing LAB strains, *L. mucosae* Q2 and *S. equinus* Q6, from sheep rumen fluid. Among these, Q6 exhibited superior enzyme production, lactic acid yield, and antimicrobial activity compared to Q2. Considering safety, acid production capacity, and antibacterial performance, Q2 shows potential as a direct-fed probiotic for livestock; whereas, Q6 demonstrates promise as a silage inoculant, but comprehensive safety assessments remain imperative for its application.

## Figures and Tables

**Table 1:** Strains most similar to the 16S rDNA sequences of isolated strains.

Strains (Accession No.)	The Highest Identity Strains (Accession No.)	Identities (%)
Q2 (PP916665)	*Lactobacillus mucosae* JCM 12515 (LC383823.1)	100
Q6 (PP916666)	*Streptococcus equinus* JCM 5802 (LC145574.1)	100
	*Streptococcus bovis* C14b1 (AF396921.1)	100

**Table 2:** Substrates fermentable by Q2 and Q6 for acid production.

Strains	Sculin	Cellobiose	Maltose	Mannitol	Salicin	Sorbitol	Sucrose	Raffinose	Inulin	Lactose
Q2	− ^1^	−	+	−	−	−	+	+	−	−
Q6	+ ^2^	+	+	−	+	+	+	+	+	+

^1^ (+) positive reaction. ^2^ (−) negative reaction.

**Table 3:** Key features of Q2 and Q6 genomes.

Features		Q2	Q6
Tatal size (bp)		2,136,340	1,950,612
Sequencing depth		156×	144×
GC content (%)		46.81	37.3
Number of contigs		2	1
Predicated gengs		2101	1928
CDSs ^1^		1984	1837
RNA genes	rRNA	24	21
	tRNA	93	70
	5S rRNA	8	7
	16S rRNA	8	7
	23S rRNA	8	7
Plasmid		1	0
ANI (%) ^2^		97.30 (*L. mucosae* DSM 13345)	97.92 (*S. equinus* ATCC 33317)

^1^ CDSs, coding sequences. ^2^ ANI, average nucleotide identity was calculated using on the Maximal Unique Matches algorithm.

**Table 4:** Number and percentage of annotated CDSs for Q2 and Q6 in universal databases.

Database	Q2 (Percentage, %)	Q6 (Percentage, %)
Nr	1957 (98.64)	1831 (99.67)
eggNOG	1669 (84.12)	1591 (86.61)
GO	1537 (77.47)	1498 (81.55)
KEGG	1080 (54.44)	1128 (61.40)
Pfam	1659 (83.62)	1645 (89.55)
Swissprot	1187 (59.83)	1231 (67.01)
TrEMBL	1963 (98.94)	1828 (99.52)
All annotated	1967 (99.14)	1831 (99.67)

Note: Nr, non-redundant protein sequence database. eggNOG, evolutionary genealogy of genes: non-supervised orthologous groups. GO, gene ontology. KEGG, Kyoto encyclopedia of genes and genomes.

**Table 5:** Annotated CDS numbers for Q2 and Q6 in special databases.

Database	Q2	Q6
CAZy	75	93
TCDB	470	501
Signal peptides	81	106
Secretory proteins	31	49
CARD 4.0	0	0
PHI-base 5.0	1	33
VFDB	0	12
dbAMP 3.0	2	4
Probiotic possibility (%)	85.82	1.44
PPRS	1.00	30.41

Note: CAZy, carbohydrate-active enzymes database. TCDB, transporter classification database. CARD, comprehensive antibiotic resistance database. PHI-base, pathogen–host interactions database. VFDB, virulence factor database. dbAMP, database of anti-microbial peptides. PPRS, probiotic potential risk score (PPRS was set as low-risk [≤4], medium-risk [4,5,6], and high-risk [≥6]).

## Data Availability

Data are contained within the article.

## Supplementary Materials

The following supporting information can be downloaded at https://www.mdpi.com/article/10.3390/microorganisms13061295/s1. Figure S1. Reads length distribution. Table S1. Basic information of sequencing data. Table S2. Genome assembly results. Table S3. Contig classification information. Table S4. Genomic components of Q2 and Q6. Table S5. The nucleotide and amino acid sequences of FAE in Q2 and Q6 genomes. Table S6. Lactate dehydrogenase genes annotated in the genomes of two strains. Table S7. Pathogenic genes predicted with TBTools-II based on the PHI-base. Table S8. Virulence factor genes predicted with TBTools-II based on the Virulence Factor Database (VFDB). Table S9. Potential antimicrobial peptides predicted with TBTools-II based on dbAMP 3.0. Table S10. Important regulatory factors that affect the production of lactic acid.

## Abbreviations

The following abbreviations are used in this manuscript:

FAE	ferulic acid esterase
LAB	lactic acid bacteria
LB	Luria-Bertani
CDSs	coding sequences
CAZy	carbohydrate-active enzymes database
FA	ferulic acid
CE	carbohydrate esterases
MOPS	3-(N-morpholino) propanesulfonic acid
MRS	The de Man, Rogosa, and Sharpe
ANI	average nucleotide identity
CRISPR	clustered regularly interspaced palindromic repeats
TCDB	Transporter Classification Database
Nr	Non-Redundant Protein Sequence Database
GO	Gene Ontology
KEGG	Kyoto Encyclopedia of Genes and Genomes
eggNOG	evolutionary genealogy of genes: Non-supervised Orthologous Groups
ARGs	antibiotic resistance genes
RGI	Resistance Gene Identifier
VFDB	virulence factor database
VFGs	virulence factor genes
AMPs	antimicrobial peptides
PGs	pathogenic genes
PPRS	probiotic potential risk score
CC	cellular component
BP	biological process
COGs	Clusters of Orthologous Groups
CAZymes	carbohydrate-active enzymes
GHs	glycoside hydrolases
GTs	glycosyl transferases
AAs	auxiliary activities
PLs	polysaccharide lyases
*ldh*	lactate dehydrogenase gene
*ldhL*	L-lactate dehydrogenase gene
*ldhD*	D-lactate dehydrogenase gene
SBSEC	*Streptococcus bovis/Streptococcus equinus* complex
BV-BRC	Bacterial and Viral Bioinformatics Resource Center
MGMT	methylated-DNA-protein-cysteine methyltransferase
frdA	flavoprotein subunit
