# Supplementary material for: Screening, Identification, and Whole-Genome Sequencing of Ferulic Acid Esterase-Producing Lactic Acid Bacteria from Sheep Rumen
Source: Microorganisms. 2025 May 31;13(6):1295. doi: 10.3390/microorganisms13061295 (PMC12194877; doi:10.3390/microorganisms13061295)
Supplement: Supplementary file 1 [file microorganisms-13-01295-s001.zip › microorganisms-3622978-supplementary.pdf]

Screening, Identification and Whole-Genome Sequencing of Ferulic Acid Esterase-Producing Lactic Acid Bacteria from Sheep Rumen

Mingxin Qiu, Yong Chen*, Lei Wang, Luyu Li, Xiao Zhang, Zhuang Ma, Jiancheng Liu*

Research Center for Biofeed and Animal Gut Health, College of Animal Science, Xinjiang Agricultural University, Urumqi 830052, China.

* *Correspondence: cy@xjau.edu.cn (Y,C.); liujc@xjau.edu.cn (J.L.)*

Supplementary material

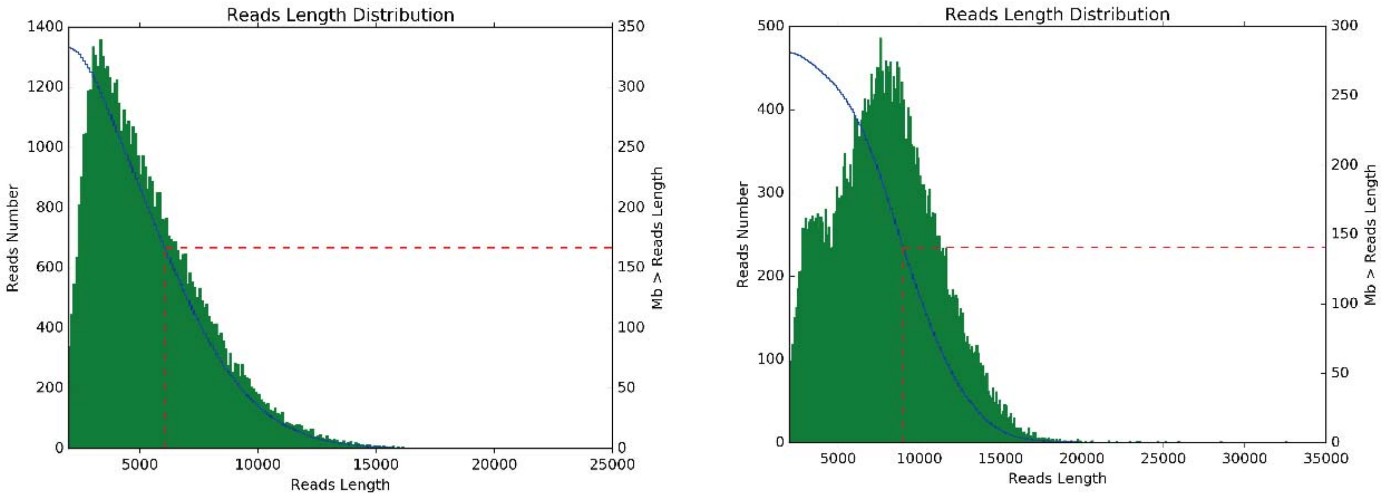

Q2 Q6

Figure S1. **Basic information of sequencing data.**

Table S1. Basic information of sequencing data

Strains	Reads	SeqNum	SumBase (bp)	N50Len (bp)	N90Len (bp)	MeanLen (bp)	MaxLen (bp)	MeanQual
Q2	Raw reads	62,700	335,650,544	6,083	3,250	5,353	21,345	34.74
	Clean reads	61,469	333,562,858	6,105	3,296	5,426	21,345	34.7
Q6	Raw reads	35,762	281,924,460	9,038	5,136	7,883	32,500	33.66
	Clean reads	35,503	281,472,346	9,045	5,162	7,928	32,500	33.64

SeqNum: Number of sequences; SumBase: Total base count; N50Len: N50 length; N90Len: N90 length; MeanLen: Mean read length; MaxLen: Maximum read length; MeanQual: Mean read quality score.

Table S2. Genome assembly results

Strains	Scaffold length (bp)	Scaffold number	Scaffold N50 (bp)	Scaffold N90 (bp)	Contig length (bp)	Contig number	Contig N50 (bp)	Contig N90 (bp)	Gaps number
Q2	2,136,340	2	2,068,302	2,068,302	2,136,340	2	2,068,302	2,068,302	0
Q6	1,950,612	1	1,950,612	1,950,612	1,950,612	1	1,950,612	1,950,612	0

Table S3. Contig classification information

Strains	Contig ID	Length	Type	Topology
Q2	Contig1	2,068,302	chromosome	circular
	Contig2	68,038	plasmid	circular
Q6	Contig1	1,950,612	chromosome	circular

Table S4. Genomic components of Q2 and Q6

Genomic component	Q2	Q6
Total repetitive sequence length (bp)	2,848	3,355
Repetitive sequence content (%)	0.13	0.17
The number of genomic islands	4	5
The number of CRISPR	13	10
The number of prophage regions	3	4
Total length of prophage (bp)	353,032	121,878
The number of gene clusters	0	3
Total length of gene cluster (bp)	0	96,003
The number of predicted promoters	552	464
The number of paralogy genes	31	2

Table S5. The nucleotide and amino acid sequences of FAE in Q2 and Q6 genomes

Strains	Loci	Nucleotide sequence	Amino acid sequence
Q2	GE000789	ATGTCAGCATCGCTCGGTGACTCGCAGCTGCTGTTAAAGACTATCAATGGTCAACAGCTGTTCGTTCGCCAAAACC<br>TACCCGTGGCGCCGGCTGCCGTATTGGTTATCGTTCATGGTCTGGGGGGGCATCAAGGTCGTTATGACTATATGAC<br>CAATTATTTCGTTCGTCACCATGTTGCCGTCTATCGCTATGATCATCGTGGTCACGGTCAGACACCTGGACCACAT<br>GGCGTTTATGGCGATTTCAATCATTTTCCTGATGATCTTAAAACCGTCGTTGATTGGGCCAAGAAAAACACGCCTC<br>ATCTGCCGATCTTTGTGGTTGGACACAGTCTGGGCGGCGGTACGGCGATGGCATTTGGCGCCAAATATCCCCAGAC<br>GGTCAATGGCATTATTTCGGTAGGAGCCTTGACGCGCTATTACCATCAGATTTTTGGTCCGGTTCGTCATTTTGGC<br>GATGATGAAACGATCAGTGGCAGCTTTGGTGATCGCAGCAACAGCAGTGCCTGGATGCGGCAAGACTATCAAGACG<br>ATCCATTGAACCTGACTGAGATTAAAGGCTCGCTGCTGAATGCGGCGATGGATCTGGTGACTTTTAATCGAACACA<br>TGCGGCCGATTTTGTCGATCCGGTCTTGATTCTGCATGGTGCCGAAGATGGCGTTGTCAGCGTCACGGATTCAATG<br>GACAGCTACCGTGAGATTTCGTCGCGCGACAAAGAGCTTCACATCTATCCATTTTTAAGACATCAGGTATTAAATG<br>AGCCATCGCGGCGGCTGGCGGTTTATCAAGAAATTTTGGACTGGATGAAAAAACATGGCTAA	MSASLGDSQLLLKTINGQQLFVRQNLPVAPA<br>AVLVIVHGLGGHQGRYDYMTNYFVRHHVAVY<br>RYDHRGHGQTPGPHGVYGDFNHFPDDLKTVV<br>DWAKKNTPHLPIFVVGHSLGGGTAMAFGAKY<br>PQTVNGIISVGALTRYYHQIFGPVRHFGDDE<br>TISGSFGDRSNSSAWMRQDYQDDPLNLTEIK<br>GSLLNAAMDLVTFNRTHAADFVDPVLILHGA<br>EDGVVSVTDSMDSYREISSRDKELHIYPFLR<br>HQVLNEPSRRLAVYQEILDWMKKHG
Q6	GE001194	ATGAAAGTTGTTGAAGTTGGTCAAGAAAATCGAGACGTTATCGTGTTGCTCCACGGTGGCGGTTTGTCCTGGTGGC<br>AGTATCAATCTCAGATGGAGCTCCTGTGTAAAAACTATCATGTGATCATTCCGATTCTTGACGGGCATGCTGGTAG<br>CAATGCTGATTTTGTCAGTATAGAAGCAAGTGCCAAGCAGCTGCTAGCTTACATCGATAAAGAATATGGTGGCTCG<br>GTTTTGCTGATTGCTGGCTTGTCGGTTGGTGGTCAGATTCTTTTAGAAATGCTTGCTTTGCGAAAAGATATTTGTC<br>AGTACGCTATTGTAGAAAGTGCTGCCATTATTTCAGATAAATTGACAGCAAGTTTGGTTGCTCCGCTATTTTCGAT<br>GAGTTTTCCTTTGATTAAGAAGAAGTGGTTTGCAAAGATGCAGTTTCGCTACCTTGGCATTCGAGCTGATTTGTTT<br>GAACATTATTATGAGGACACGGTGAAACTTTCTAAGCAAAATTTGATTGCTTTTACAAAAGCAAGTAGCTTGTATC<br>AAGTAAAGAAAAATTTGAAGAATTCGTTAGCGCGTGTTCGCATTATTGTGGGAGAAAAAGAAACTAAAAAGATGCA<br>CGCTTCAGCTAAAATGCTTCACGATATTTTGCCAGACAGCCATCTTGAAATCAAATCTGGTCTAGCTCACGGTCAG<br>TATTCTATTAATTATCCAGAATTATATGTCAAAGAACTTCTGGAGGAAATTCAAAGAAGTGAAATCTAA	MKVVEVGQENRDVIVLLHGGGLSWWQYQSQM<br>ELLCKNYHVIIPILDGHAGSNADFVSIEASA<br>KQLLAYIDKEYGGSVLLIAGLSVGGQILLEM<br>LALRKDICQYAIVESAAIISDKLTASLVAPL<br>FSMSFPLIKKKWFAKMQFRYLGIRADLFEHY<br>YEDTVKLSKQNLIAFTKASSLYQVKKNLKNS<br>LARVRIIVGEKETKKMHASAKMLHDILPDSH<br>LEIKSGLAHGQYSINYPELYVKELLEEIQRS<br>EI

Table S6. Lactate dehydrogenase genes annotated in the genomes of two strains

Strains	Loci	KEGG annotation	Nr annotation
Q2	GE000312	K00016|L-lactate dehydrogenase	'L-lactate dehydrogenase [Lactobacillus mucosae]', 'gi|493546594|ref|WP_006500413.1|', '2.1e-173', '100.00', '100.00', '617']
Q2	GE000706	K03778|D-lactate dehydrogenase	'D-2-hydroxyacid dehydrogenase [Lactobacillus mucosae]', 'gi|1561218421|ref|WP_128513234.1|', '5.9e-187', '99.70', '100.00', '662']
Q2	GE000707	K03778|D-lactate dehydrogenase	'D-2-hydroxyacid dehydrogenase [Lactobacillus mucosae]', 'gi|729053964|ref|WP_033935050.1|', '1.4e-188', '100.00', '100.00', '667']
Q2	GE000747	K00016|L-lactate dehydrogenase	'L-lactate dehydrogenase [Lactobacillus mucosae]', 'gi|729054554|ref|WP_033935623.1|', '5.7e-171', '100.00', '100.00', '609']
Q2	GE001003	K00016|L-lactate dehydrogenase	'L-lactate dehydrogenase [Lactobacillus mucosae]', 'gi|729054470|ref|WP_033935543.1|', '2.1e-176', '100.00', '100.00', '627']
Q2	GE001115	K03778|D-lactate dehydrogenase	'D-2-hydroxyacid dehydrogenase [Lactobacillus mucosae]', 'gi|748687581|ref|WP_039945705.1|', '6.8e-183', '100.00', '100.00', '649']
Q2	GE001737	K00016|L-lactate dehydrogenase	'L-lactate dehydrogenase [Lactobacillus mucosae]', 'gi|1561217810|ref|WP_128512812.1|', '2.0e-165', '100.00', '100.00', '590']
Q2	GE001805	K00016|L-lactate dehydrogenase	'L-lactate dehydrogenase [Lactobacillus mucosae]', 'gi|736526252|ref|WP_034541190.1|', '7.9e-173', '100.00', '100.00', '615']
Q6	GE000927	K00016|L-lactate dehydrogenase	'L-lactate dehydrogenase [Streptococcus equinus]', 'gi|636817946|ref|WP_024344063.1|', '2.4e-180', '100.00', '100.00', '640']
Q6	GE001121	K00016|L-lactate dehydrogenase	'L-lactate dehydrogenase [Streptococcus equinus]', 'gi|636817780|ref|WP_024343897.1|', '3.7e-167', '99.34', '100.00', '596']

Table S7. Pathogenic genes predicted with TBTools-Ⅱ based on the PHI-base

Strains	Loci	Pathogenic genes				Identity (%)	Coverage (%)	E value	Location
		Protein ID	Gene name	Function	Mutant phenotype				
Q2	GE000015	S4E4Q5	WalR	Transcriptional regulatory protein	increased virulence (hypervirulence)	80.60	98.72	3.43e-135	chromosome
Q6	GE000020	Q1J943	prsA2	Ribose-phosphate pyrophosphokinase	reduced virulence	94.36	98.15	0	chromosome
Q6	GE000223	P0C0B9	bglB	Glycosyltransferase known to attach GlcNAc to SRRPs	reduced virulence	93.96	99.67	0	chromosome
Q6	GE000291	P66719	RopE	putative DNA-directed RNA polymerase (delta subunit)	reduced virulence	82.46	90.00	5.00E-94	chromosome
Q6	GE000301	Q1JLQ2	MntE	Ketol-acid reductoisomerase (NADP (+))	reduced virulence	92.74	99.44	0	chromosome
Q6	GE000362	Q99Z66	ptsI	essential gene	lethal	92.2	99.83	5.84E-61	chromosome
Q6	GE000388	Q8DYL6	uvrB	essential gene	lethal	90.35	99.85	1.29E-46	chromosome
Q6	GE000476	Q8DPC2	potA	Manganese transporter periplasmic protein	unaffected pathogenicity	89	98.96	0	prophage_region 1
Q6	GE000612	D5AF41	IMPDH	Phosphoenolpyruvate-protein phosphotransferase	increased virulence (hypervirulence)	88.03	99.80	0	chromosome
Q6	GE000744	B5TQV9	PGM	Central tRNA-modifying GTPase	reduced virulence	86.69	99.48	0	chromosome
Q6	GE000750	A4VWH9	Eno	Transcription facot	reduced virulence	85.52	99.31	0	chromosome
Q6	GE000770	A4VYE0	gidA	dTDP-4-dehydrorhamnose reductas	lethal	85.01	98.12	1.96E-178	chromosome
Q6	GE000825	P0A452	recA	Redox-sensing transcriptional repressor	reduced virulence	84.42	98.71	1.88E-136	prophage region 2
Q6	GE000850	Q97RW0	SP_0676	Manganese Homeostasis	unaffected pathogenicity	84.05	93.19	0	chromosome
Q6	GE000921	A4VWD1	mnmE	phosphoglucomutase	reduced virulence	83.37	99.78	0	chromosome
Q6	GE000927	P0A3M9	ldh	L-lactate dehydrogenase	reduced virulence	89.67	100.00	0	chromosome
Q6	GE000938	A0A0M9FFE8	ciaR	DNA-binding response regulator	reduced virulence	89.69	100.00	2.00E-152	chromosome
Q6	GE000953	A0A0H2URT4	gtfA	Transcriptional regulator	reduced virulence	81.88	99.79	5.56E-87	chromosome

Q6	GE001002	A0A2J9QCW3	dltA	ABC transporter ATP-binding protein - spermidine/putrescine transport	reduced virulence	81.21	98.65	0	chromosome
Q6	GE001168	Q1JJJ3	PepO	Excinuclease ABC, B subunit	unaffected pathogenicity	80.51	99.84	0	chromosome
Q6	GE001217	Q9A0G6	GacA	Enolase	unaffected pathogenicity	83.1	99.65	0	chromosome
Q6	GE001330	A0A3L8GGZ3	MurI	Responder protein	unaffected pathogenicity	81.44	99.62	4.81E-159	chromosome
Q6	GE001366	Q93MY4	covR	Putative phospho--glucosidase	increased virulence (hypervirulence)	90.68	99.58	0	chromosome
Q6	GE001457	P0A4H7	CiaR	Glutamate racemase	reduced virulence	87.5	99.56	1.63E-165	chromosome
Q6	GE001474	Q8E565	gbs1167	regulator	unaffected pathogenicity	84.29	98.59	3.47E-103	chromosome
Q6	GE001587	A0A0H2UNL5	pfl2	formate acetyltransferase	reduced virulence	91.71	100.00	0	chromosome
Q6	GE001672	P67320	nrdR	D-alanine--poly(phosphoribitol) ligase	reduced virulence	85.37	99.39	0	chromosome
Q6	GE001741	U2W9V3	SpxA2	Endopeptidase	reduced virulence	92.42	99.25	0	chromosome
Q6	GE001754	U2UML5	SpxA1	essential gene	lethal	86.57	99.26	2.81E-51	chromosome
Q6	GE001756	Q8DQV4	spr0479	pathogenitcity related	reduced virulence	90.62	97.96	1.13E-54	chromosome
Q6	GE001757	Q8CZ89	spr0175	Transcriptional regulator	increased virulence (hypervirulence)	88.64	98.88	1.94E-89	chromosome
Q6	GE001758	Q8DRE1	spr0177	DNA recombination and repair	reduced virulence	85.23	86.27	0	chromosome
Q6	GE001813	Q8DP67	spr1327	tRNA uridine 5-carboxymethylaminomethyl modification enzyme	reduced virulence	81.18	98.84	0	chromosome
Q6	GE001830	Q04M32	ilvC	Inosine-5'-monophosphate dehydrogenase	reduced virulence	89.12	99.71	0	chromosome

BLASTP v2.8.1+ (Database: PHI-base v4.14, Nov. 2022 release)

Table S8. Virulence Factor genes predicted with TBTools-Ⅱ based on the Virulence Factor Database (VFDB)

Strains	Loci	Virulence Factors						Identity (%)	Coverage (%)	E value	Location
		Gene ID	Gene name	VF name	VF category	Accession no.	Product				
Q6	GE000136	VFG005766	cylG	β-haemolysin/cytolysin	Exotoxin	WP_000861302	3-ketoacyl-ACP-reductase CylG	80.00	100	1.67E-150	chromosome
Q6	GE000137	VFG005769	csuA	Csu fimbriae	Biofilm	WP_000611493	Csu pilus subunit	81.44	97	7.00E-59	chromosome
Q6	GE000138	VFG005772	cylZ	β-haemolysin/cytolysin	Exotoxin	WP_000164166	3R-hydroxymyristoyl ACP dehydratase	80.38	100	2.00E-99	chromosome
Q6	GE000290	VFG005546	tig/ropA	Trigger factor	Stress survival	WP_000107753	trigger factor	88.06	100	0	chromosome
Q6	GE000669	VFG005898	rfbA	Capsule	Immune modulation	WP_002904719	glucose-1-phosphate thymidylyltransferase RfbA	90.66	100	0	chromosome
Q6	GE000670	VFG005985	/	Capsule	Immune modulation	WP_002263085	dTDP-4-dehydrorhamnose 3,5-epimerase family protein	93.33	98	6E-141	chromosome
Q6	GE000671	VFG006062	rfbB	Capsule	Immune modulation	WP_002947383	dTDP-glucose 4,6-dehydratase	95.68	100	0	chromosome
Q6	GE000750	VFG006042	/	Capsule	Immune modulation	WP_001222601	LysR family transcriptional regulator	83.39	100	0	chromosome
Q6	GE000770	VFG005889	rfbD	Capsule	Immune modulation	WP_002261974	dTDP-4-dehydrorhamnose reductase	90.49	100	0	chromosome
Q6	GE001217	VFG005579	eno	Streptococcal enolase	Exoenzyme	WP_000022815	phosphopyruvate hydratase	93.09	100	0	chromosome

Q6	GE001621	VFG005360	plr/gapA	Streptococcal plasmin receptor/GAPDH	Adherence	WP_002262489	type I glyceraldehyde-3-phosphate dehydrogenase	85.21	100	0	genomic_island 4
Q6	GE001712	VFG005865	galU	Capsule	Immune modulation	WP_002262524	UTP--glucose-1-phosphate uridylyltransferase	87.87	100	0	chromosome

BLASTN v2.8.1+ (Database: VFDB, Mar. 2025 release)

Table S9. Potential antimicrobial peptides predicted with TBTools-Ⅱ based on dbAMP 3.0

Strains	Loci	Query sequences	Target	Target sequences	Identity (%)	E value	Bit score	Coverage (%)
Q2	GE000761	MSKTIVRKNESLDDALRRFKRTVSRNGTLQEYRKREFYEKPSVKRKLKSEAARKRKNKRRH	dbAMP_26911	SKTIVRKNESIDDALRRFKRAVSKTGTLQEVRKREFYEKPSVRRKKKSEAARKRK	87.27	1.32E-28	93.60	90.00
Q2	GE000805	MANKAQLVSDVATATGLTKKDATAAVDAVFSSIQASLAKGEKVQLIGFGNFEVRQRAARKGRNPQTGQEIEIPASKVPAFKPGKALKDAVK	dbAMP_32714	MANKAELIDSVASKTGLTKKDATSAVDAVFETIQENLSEGNKVQLIGFGNFEVRQRAARKGRNPQTGEEIKIPASKVPAFKPGKALKDSVK	81.32	6.09E-52	154.00	100.00
Q6	GE000048	MGRSLKKGPFVDEHLMKKVEAQANDEKKKVIKTWSRRSTIFPSFIGYTIAVYDGRKHVPVYIQEDMVGHKLGEFAPTRTYKGHAADDKKTR	dbAMP_25710	ARSLKKGPFVDGHLMTKIEKLNETDKKQVVKTWSRRSTIFPQFIGHTIAVYDGRKHVPVFISEDMVGHKLGEFAPTRTYKGHASDDKKTRR	81.11	1.67E-52	156.00	99.00
Q6	GE000053	MERNQRKTLVGRVVSNKMDKTITVVVETKRNHPVYGKRINYSKKYKAHDENNVAKEGDIVRIMETRPLSATKRFRLVEVVEKAVII	dbAMP_26899	SERNQRKVYVGRVVSDKMDKTITVLVETYKKHPLYGKRVKYSKKYKAHDEHNEAKVGDIVKIMETRPLSATKRFRLVEIVEKAVVL	80.00	4.84E-47	142.00	99.00
Q6	GE000057	MAKKSMIAKNHRPAKFSTQAYTRCERCGRPHSVYRKFKLCRVCFRELAYKGQIPGVTKASW	dbAMP_25682	AKKSMIAKQQRTPKFKVQEYTRCERCGRPHSVIRKFKLCRICFRELAYKGQIPGVKKASW	83.33	1.34E-33	106.00	98.00
Q6	GE001156	MSKTVVRKNESLDDALRRFKRSVTKAGTLQESRKREFYEKPSVKRKRKSEAARKRKKF	dbAMP_26912	SKTVVRKNESLEDALRRFKRSVSKTGTLQEARKREFYEKPSVKRKKKSEAARKRKF	90.91	8.43E-30	96.70	95.00

Table S10. Important regulatory factors that affect the production of lactic acid

Strains	Loci	Effect	Factors	KEGG annotation	Nr annotation
Q2	GE000492	Increase	glutamate racemase	K01776|glutamate racemase	'glutamate racemase [Lactobacillus mucosae]', 'gi|729053348|ref|WP_033934443.1|', '3.7e-147', '99.62', '100.00', '530']
Q2	GE001589	Increase	Methylated-DNA--protein-cysteine methyltransferase	K00567|methylated-DNA-[protein]-cysteine S-methyltransferase	'6-O-methylguanine DNA methyltransferase [Lactobacillus mucosae LM1]', 'gi|767527832|ref|AJT49973.1|', '9.9e-90', '97.60', '100.00', '338']
Q2	GE001738	Increase	Fumarate reductase flavoprotein subunit	K00244|fumarate reductase flavoprotein subunit	'flavocytochrome c [Lactobacillus mucosae]', 'gi|856993794|ref|WP_048345774.1|', '4.9e-264', '100.00', '100.00', '919']
Q2	GE001245	Increase	Fumarate reductase flavoprotein subunit	--	'FAD-binding protein [Lactobacillus mucosae]', 'gi|729053794|ref|WP_033934885.1|', '0.0e+00', '99.67', '100.00', '1222']
Q2	GE000115	Increase	Amidophosphoribosyltran sferase	K00764|amidophosphoribosyl transferase	'amidophosphoribosyltransferase [Lactobacillus mucosae]', 'gi|1561218662|ref|WP_128513391.1|', '3.2e-282', '99.39', '100.00', '979']
Q2	GE000904	Decrease	Ribokinase	--	'ribokinase [Lactobacillus mucosae]', 'gi|1561218655|ref|WP_128513386.1|', '2.6e-170', '100.00', '100.00', '607']
Q2	GE001491	Decrease	Ribokinase	K00852|ribokinase	'ribokinase [Lactobacillus mucosae]', 'gi|729053400|ref|WP_033934494.1|', '4.2e-163', '100.00', '100.00', '583']
Q2	GE001820	Decrease	Ribokinase	K00852|ribokinase	'ribokinase [Lactobacillus mucosae]', 'gi|736526240|ref|WP_034541182.1|', '6.3e-167', '100.00', '100.00', '595']
Q2	GE001935	Decrease	Ribokinase	K00852|ribokinase	'ribokinase [Lactobacillus mucosae]', 'gi|729053744|ref|WP_033934836.1|', '5.9e-165', '99.02', '100.00', '589']
Q2	GE001111	Decrease	Fructokinase	K00847|fructokinase	'ROK family protein [Lactobacillus mucosae]', 'gi|729054354|ref|WP_033935430.1|', '4.0e-171', '100.00', '100.00', '609']
Q2	GE001231	Decrease	Fructokinase	K00847|fructokinase	'ROK family protein [Lactobacillus mucosae]', 'gi|493545381|ref|WP_006499220.1|', '3.9e-174', '100.00', '100.00', '619']
Q2	GE000024	Decrease	Argininosuccinate lyase	K01755|argininosuccinate lyase	'argininosuccinate lyase [Lactobacillus mucosae]', 'gi|856993678|ref|WP_048345658.1|', '2.5e-260', '100.00', '100.00', '906']
Q2	GE001661	Decrease	large subunit ribosomal protein L30	K02907|large subunit ribosomal protein L30	'50S ribosomal protein L30 [Lactobacillus mucosae]', 'gi|493546791|ref|WP_006500605.1|', '2.9e-23', '100.00', '100.00', '116']
Q6	GE001457	Increase	glutamate racemase	K01776|glutamate racemase	'glutamate racemase [Streptococcus equinus]', 'gi|654498644|ref|WP_027968415.1|', '3.6e-150', '100.00', '100.00', '540']

Q6	GE001564	Increase	Fumarate reductase flavoprotein subunit	K00244|fumarate reductase flavoprotein subunit	'flavocytochrome c [Streptococcus equinus]', 'gi|636817451|ref|WP_024343568.1|', '0.0e+00', '99.63', '100.00', '1587']
Q6	GE000027	Increase	Amidophosphoribosyltransferase	K00764|amidophosphoribosyl transferase	'amidophosphoribosyltransferase [Streptococcus equinus]', 'gi|544711374|ref|WP_021141388.1|', '4.1e-285', '100.00', '100.00', '989']
Q6	GE001535	Decrease	fructokinase	K00847|fructokinase	'ROK family protein [Streptococcus equinus]', 'gi|544713060|ref|WP_021143074.1|', '3.2e-168', '100.00', '100.00', '600']
Q6	GE001697	Decrease	argininosuccinate lyase	K01755|argininosuccinate lyase	'argininosuccinate lyase [Streptococcus equinus]', 'gi|1086106408|ref|SDI83332.1|', '8.7e-269', '99.79', '100.00', '934']
Q6	GE000062	Decrease	large subunit ribosomal protein L30	K02907|large subunit ribosomal protein L30	'MULTISPECIES: 50S ribosomal protein L30 [Streptococcus]', 'gi|493578224|ref|WP_006531331.1|', '1.4e-22', '100.00', '100.00', '114']
